# Looping Mediated Interaction between the Promoter and 3′ UTR Regulates Type II Collagen Expression in Chondrocytes

**DOI:** 10.1371/journal.pone.0040828

**Published:** 2012-07-16

**Authors:** Arijita Jash, Kangsun Yun, Anupama Sahoo, Jae-Seon So, Sin-Hyeog Im

**Affiliations:** School of Life Sciences and Immune Synapse Research Center, Gwangju Institute of Science and Technology (GIST), Gwangju, Korea; University of Saarland Medical School, Germany

## Abstract

Type II collagen is the major component of articular cartilage and is mainly synthesized by chondrocytes. Repeated sub-culturing of primary chondrocytes leads to reduction of type II collagen gene (*Col2a1*) expression, which mimics the process of chondrocyte dedifferentiation. Although the functional importance of *Col2a1* expression has been extensively investigated, mechanism of transcriptional regulation during chondrocyte dedifferentiation is still unclear. In this study, we have investigated the crosstalk between *cis*-acting DNA element and transcription factor on *Col2a1* expression in primary chondrocytes. Bioinformatic analysis revealed the potential regulatory regions in the *Col2a1* genomic locus. Among them, promoter and 3′ untranslated region (UTR) showed highly accessible chromatin architecture with enriched recruitment of active chromatin markers in primary chondrocytes. 3′ UTR has a potent enhancer function which recruits Lef1 (Lymphoid enhancer binding factor 1) transcription factor, leading to juxtaposition of the 3′ UTR with the promoter through gene looping resulting in up-regulation of *Col2a1* gene transcription. Knock-down of endogenous Lef1 level significantly reduced the gene looping and subsequently down-regulated *Col2a1* expression. However, these regulatory loci become inaccessible due to condensed chromatin architecture as chondrocytes dedifferentiate which was accompanied by a reduction of gene looping and down-regulation of *Col2a1* expression. Our results indicate that Lef1 mediated looping between promoter and 3′ UTR under the permissive chromatin architecture upregulates *Col2a1* expression in primary chondrocytes.

## Introduction

Type II collagen is the major collagen synthesized by chondrocytes in articular cartilage and forms an integral component of extracellular matrix (ECM) [1], [2]. Along with collagen IX and XI, it forms the fibrillar collagen network which is responsible for providing tensile strength to the cartilage tissue, thereby protecting the underlying bone from mechanical injury and damage. Disrupted expression of type II collagen features essentially in bone and cartilage degenerative diseases such as spondyloepiphyseal dysplasia, achondrogenesis type II, Stickler syndrome and Kniest dysplasia [3], [4], [5], [6]. These reports suggest that type II collagen can serve as a marker of healthy cartilage and understanding the regulation mechanism of its expression will contribute to develop novel therapeutics for cartilage and bone degenerative diseases like osteoarthritis and rheumatoid arthritis [7], [8], [9].

Type II collagen is transcribed at a high rate in the chondrocytes that are also responsible for producing many types of ECM proteins. These cells following isolation and regular sub culturing in monolayer gradually lose their phenotype and adapt a flattened fibroblast like phenotype with a significant decrease in type II collagen gene (*Col2a1*) expression [10]. Thus chondrocytes exhibit two distinct stages in the context of type II collagen expression - a state of robust expression, followed by a stage with almost no expression observed at the level of transcription**.** This indicates the existence of a balanced interaction between positive and negative factors that govern the expression of type II collagen. *Col2a1* locus encompasses around 50 kb of chromosome 12 and 15 in case of human and mouse respectively. The gene spans a region of about 30 kb from the TSS (transcription start site) to the polyadenylation site. A conserved promoter with a TATA box, GC box but no CAAT box generates a major *Col2a1* transcript of 5 kb that is translated into a polypeptide with approximately 1,400 amino acids. Coding sequences of mouse and human type II collagen are 89% identical at the nucleotide level and in the mature polypeptide 37 amino acids are altered. The gene locus contains numerous conserved non-coding sequences (CNSs) which are also highly conserved between human and mouse [11]. CNSs in general, are known to serve as platforms for recruiting transcription factors that help in gene transcription by the RNA polymerase II (Pol II) machinery [12]. Furthermore, CNSs also undergo epigenetic modifications like DNA methylation and histone modifications. These modifications often influence the chromatin structure thereby governing transcriptional status of gene expression through coordinated recruitment of specific transcription factors [13], [14], [15], [16], [17]. CNS mediated gene expression can occur locally and over large genomic distances as they are frequently positioned far upstream or downstream of the genes. CNSs control and interact with their target promoters efficiently in the presence of a unique combination of enhancer binding proteins, a couple of which can bend or loop the DNA to facilitate intra- and inter-chromosomal interactions [18], [19]. Interestingly, however, in spite of the presence of numerous CNSs in the *Col2a1* locus and their high rate of conservancy between mouse and human, only the first intron so far has been identified to function as an enhancer [8], [20], [21], [22], [23].

In this study, we identified a novel function of 3′ UTR as a key regulatory element to enhance *Col2a1* transcription. *In vivo* binding of Lef1 to the 3′ UTR induced a DNA looping interaction between the *Col2a1* promoter and 3′ UTR thereby potentiating *Col2a1* transcription. Significant reduction of Lef1 binding to the 3′ UTR during chondrocyte dedifferentiation was well correlated with decreased *Col2a1* expression. We suggest that Lef1 binding to the 3′ UTR under the epigenetically permissive chromatin architecture upregulates type II collagen expression through DNA looping in primary chondrocytes.

## Results

### Identification of Potential Regulatory Element in the *Col2a1* Locus

**Figure 1:**
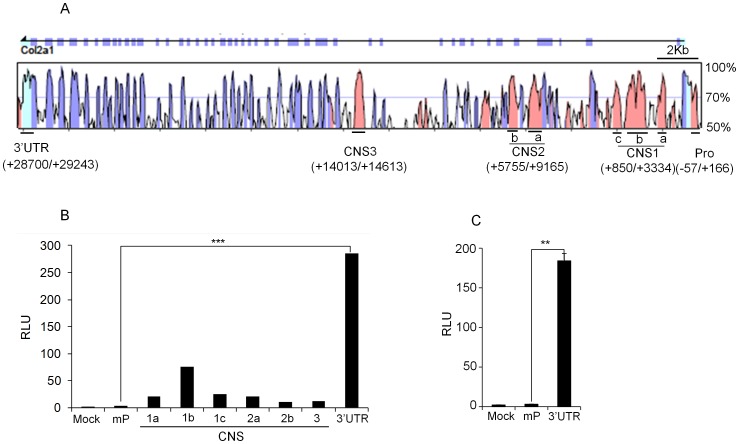
Identification of regulatory elements in the *Col2a1* locus. (A) Conserved noncoding sequences (CNSs) in the *Col2a1* loci of mouse and human are shown. rVISTA 2.0 analysis depicting % conservation between mouse (as base) and human *Col2a1* loci. Peaks in pink color indicate the intronic regions, violet peaks indicate the exons and sky blue peaks indicate the 5′ and 3′ UTRs, respectively. The relative positions of CNS regions are named according to their distance in kb from the transcription start site. (B) HTB-94 human chondrosarcoma cells were transfected with the luciferase reporter alone (mock), construct containing the *Col2a1* minimal promoter only (mP) or with the constructs containing the individual CNS regions with the minimal promoter, and luciferase activity was measured 24 hrs post transfection. (C) Primary mouse chondrocytes were transfected with indicated luciferase reporter plasmids and luciferase activity was measured 24 hrs post transfection. In each case, plasmid encoding Renilla luciferase (pRLTK) was used as normalization control and data was expressed relative to the activity of Renilla luciferase as Relative Luciferase Unit (RLU). The data shown are expressed as mean ± SEM, n = 3 and **P<0.01, ***P<0.001.

To identify candidate *cis*-acting regulatory elements in the *Col2a1* locus, bioinformatic analysis was performed using the web-based global alignment tool VISTA [24]. Mouse *Col2a1* locus was aligned with the sequence of human based on the degree of conservancy between the two species. We identified five CNSs (conserved non-coding sequences), defined as having 75% or greater identity over at least 100 bp stretches upstream and downstream of the *Col2a1* locus (Fig. 1A). These include the minimal promoter (−57/+166), CNS1 (+850/+3334), CNS2 (+5755/+9165), CNS3 (+14013/+14613) and 3′ UTR (+28700/+29243) (Fig. 1A). To investigate if any of these CNSs could enhance *Col2a1* expression, we made luciferase reporter constructs and examined their enhancer activity in HTB-94 human chondrosarcoma cell line. Col2a1 minimal promoter (mP) was cloned into pXPG luciferase vector [21] and used as a basal *cis*-acting element for *Col2a1* transcription. Into this basic construct, each CNS fragment was cloned into the upstream of the minimal promoter (Fig. 1B). Luciferase assay revealed that the reporter activity of the 3′ UTR (549 bp) was more than 270-fold higher than the level of mP in HTB-94 human chondrosarcoma cells (Fig. 1B). The other CNS reporter constructs except CNS 1b (+2014/+2405) [25], showed marginal effects on transcriptional activity. We further tested whether the 3′ UTR also has enhancer activity in primary chondrocytes. Upon transfection into primary mouse chondrocytes 3′ UTR also showed high transactivity (Fig. 1C). These results suggest that the previously uncharacterized 3′ UTR may have important regulatory roles in *Col2a1* expression in chondrocytes.

### The 3′ UTR Acts as an Enhancer to Up-regulate *Col2a1* Transcription

**Figure 2:**
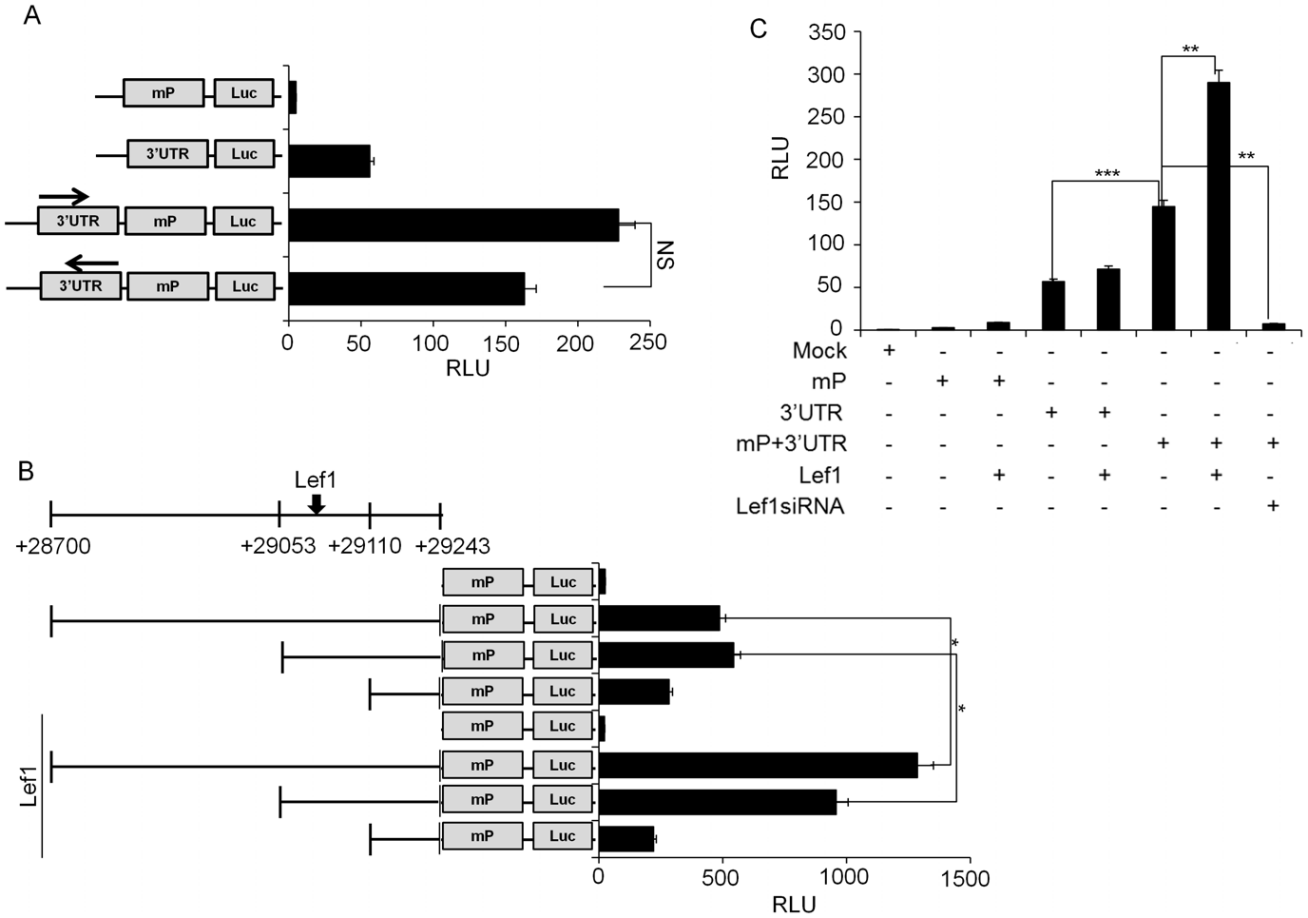
The 3′ UTR has enhancer activity. (A) HTB-94 human chondrosarcoma cells were transfected with empty luciferase reporter plasmid (mock) or luciferase reporter constructs containing *Col2a1* minimal promoter, 3′ UTR alone, 3′ UTR in conjunction with *Col2a1* minimal promoter in right or reverse orientation. (B) Various 3′ UTR reporter constructs (full length (28699/+29243), 190 bp (+29053/+29243) and 134 bp (+29109/+29243)), were transfected into HTB-94 human chondrosarcoma cells along with Lef1 expression plasmid and luciferase activity was measured. (C) Lef1 dependent increase of enhancer activity of the 3′ UTR was verified in primary chondrocytes. In all experiments, luciferase activity is expressed relative to the expression of a co-transfected Renilla luciferase plasmid (pRL-TK) as a control for transfection efficiency. NS. Not significant. All data are representative of three independent experiments. *P<0.05, **P<0.01.

To check whether the 3′ UTR has basal promoter or enhancer activity, we cloned 3′ UTR region (+28700/+29243) and performed transient reporter assay. Luciferase assay with the 3′ UTR alone or in conjunction with the minimal promoter (−57/+166) was performed in HTB-94 human chondrosarcoma cell line. The 3′ UTR alone has basal promoter activity and showed a strong enhancer activity upon coupling with the *Col2a1* minimal promoter (Fig. 2A). To check whether the enhancer activity of the 3′ UTR is independent of its orientation, we cloned it in the reverse direction and found similar transactivity of 3′ UTR as in right orientation (Fig. 2A).

To further identify the key regulatory region within the 3′ UTR and transcription factors that bind to the locus, we performed luciferase assay with a series of 3′ UTR deletion constructs. High levels of transactivity of the 3′ UTR is maintained until the length of 189 bp (+29054/+29243). However, removal of another 56 bp to a size of 133 bp (+29110/+29243) results in a significant loss of its activity. Analysis of the genomic sequence of 3′ UTR by TRNASFAC database revealed a location of conserved Lef1 binding site within the 56 bp region (Fig. S1). Thus, we tested the effect of Lef1 over-expression on 3′ UTR driven transactivity using various 3′ UTR deletion constructs such as full length (28700/+29243), 189 bp (+29054/+29243) and 133 bp (+29110/+29243). Lef1 over-expression significantly increased the transactivity of both the full length (28700/+29243) and 189 bp 3′ UTR (+29054/+29243). However 133 bp 3′ UTR (+29110/+29243) construct failed to transactivate the promoter activity, which is caused by the deletion of Lef1 binding site (Fig. 2B). Lef1 over-expression also enhanced the transactivity of the full length 3′ UTR in primary chondrocytes and cotransfection of Lef1 siRNA significantly decreased the inherent high activity of the 3′ UTR (Fig. 2C).

### Lef1 Binding to the 3′ UTR Regulates *Col2a1* Expression

**Figure 3:**
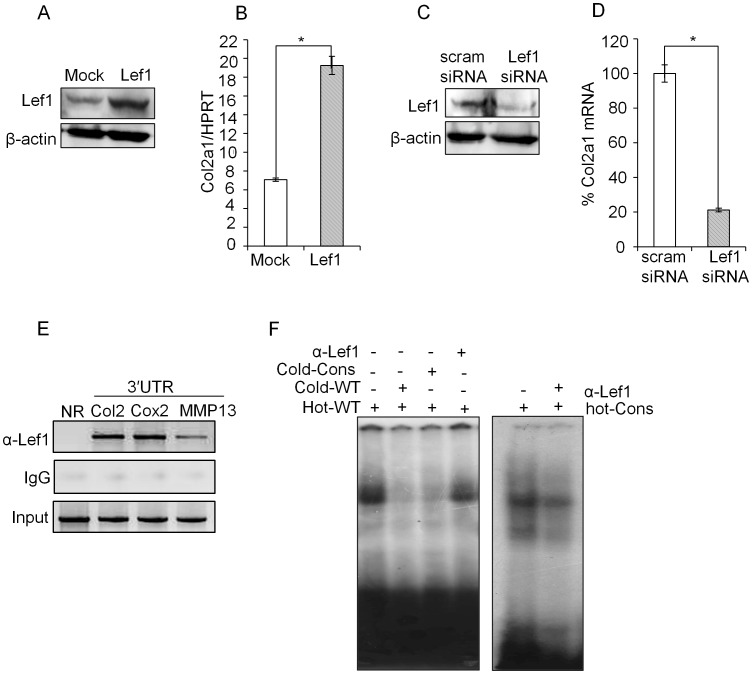
Lef1 dependent *Col2a1* expression in primary chondrocytes. (A, B). Primary chondrocytes were transfected with Lef1 expression plasmid or empty vector control (mock). Lef1 over-expression was confirmed by immunoblotting with antibodies against Lef1 and β-actin (control) and relative level *of Col2a1* transcript was detected by qRT-PCR and expressed relative to the level of housekeeping control HPRT. (C, D) Primary chondrocytes were transfected with scrambled siRNA (mock) or Lef1 siRNA and Lef1 knock-down was confirmed by immunoblotting and relative level of *Col2a1* transcript was detected by qRT-PCR and expressed relative to the level of housekeeping control HPRT. Knock-down efficiency was expressed as percentage of scrambled siRNA (control) transfected sample. (E) Physiological binding of Lef1 to the predicted conserved Lef1-binding site in the 3′ UTR was assessed by ChIP assay. Crosslinked and fragmented DNA from primary chondrocytes were immunoprecipitated with Lef1 antibody and IgG (control). PCR analysis was performed using the primers for the 3′ UTR region of *Col2a1* locus. The same precipitate was also probed with primers specific for the 3′ UTR regions of Cox2 and MMP13 (positive controls) [27], [28], [29], [30] or primers specific for the non-conserved region (+16801/+17024) in the *Col2a1* locus (negative control). All data are representative of three independent experiments. *P<0.05, **P<0.01. (F) EMSA was performed by incubating nuclear extract prepared from P0 stage chondrocyte with the indicated labeled probes, (hot-wild type (hot-WT) and hot-consensus (hot-Cons) and non-labeled competitor oligonucleotides (cold-wild type (cold-WT) and cold-Consensus (cold-Cons) or α-Lef1 antibody.

To further confirm the functional importance of Lef1 we tested the effect of Lef1 over-expression or knock-down on *Col2a1* expression in primary chondrocytes. Lef1 expression plasmid or Lef1 siRNA was transfected into primary chondrocytes and Col2a1 expression was measured by qRT-PCR. Lef1 over-expression (Fig. 3A) significantly increased *Col2a1* expression (Fig. 3B), while siRNA mediated knock-down of Lef1 (Fig. 3C) significantly decreased *Col2a1* expression (Fig. 3D). As a control, the level of Lef1 protein upon transfection of Lef1 expression vector (Fig. 3A) or siRNA (Fig. 3A) was measured by western blot analysis.

To test whether interaction of Lef1 to the 3′ UTR is occurring *in vivo* we performed chromatin immunoprecipitation assay (ChIP) and confirmed its binding to the 3′ UTR (Fig. 3E). Additionally, to further confirm the direct binding of Lef1 to the 3′ UTR, EMSA was performed. Incubation of the total cell extract from chondrocytes with Lef1 binding site containing probe (Hot-WT), corresponding to the 3′ UTR, resulted in a formation of high intensity complex (lane 1 in Fig. 3F). However, addition of non-radio labeled Lef1 probe (Cold-WT) or with a Lef1 consensus probe (Cold-Cons) [26] significantly decreased the intensity. Moreover, addition of Lef1 antibody also decreased the intensity of the complex (lane 4 in left panel and lane 2 right panel in Fig. 3F). These results demonstrated that the Lef1 binding of 3′ UTR is closely linked with *Col2a1* expression in chondrocytes.

### Lef1 Induced Gene Looping Mediates Physical Interaction of 3′ UTR with the Promoter Region

**Figure 4:**
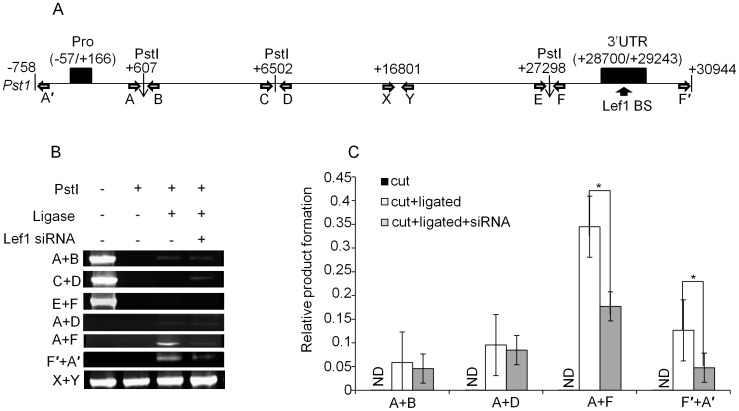
Juxtaposition of 3′ UTR with the promoter through gene looping. (A) The relative position of the primers and *Pst1* cleavage sites designed to detect gene looping between the Col2a1 promoter and 3′ UTR are denoted as described in the main text and Fig. 1A. Figure was not drawn in scale. The filled black boxes indicate the promoter and 3′ UTR. X and Y denote primer pairs that do not flank any *Pst1* site. The empty horizontal arrows denote the 3C primers and the vertical black arrow indicates the Lef1 binding site (BS) in the 3′ UTR. (B, C) 3C assay was performed. Nuclei prepared from uncrosslinked (Fig. S2), formaldehyde crosslinked or Lef1 siRNA transfected primary chondrocytes were subjected to *Pst1* digestion. Digestion efficiency was confirmed by RT-PCR using primers flanking the *Pst1* sites (Fig. S2). The formation of 3C product was detected by qRT-PCR using digested (cut), digested and ligated (cut + ligated) or Lef1 siRNA treated, digested and ligated (cut + ligated + siRNA) DNA samples as templates. The results are shown as EtBr stained gel images (B) or quantitative representation (C) of the product obtained with the indicated primer pairs relative to that obtained with control primer pairs X+Y. The data shown are expressed as mean ± SEM, n = 3 and *P<0.05.

Architectural transcription factors can bend the distal regulatory regions to juxtapose it with the promoter or other *cis*-acting elements. We and other groups have previously demonstrated that Lef1 can regulate its target genes such as Cox2 and MMP13 through gene looping [27], [28], [29], [30]. In this study, we also tested the possibility that Lef1 binding to 3′ UTR can induce DNA looping to juxtapose 3′ UTR with the *Col2a1* promoter by performing chromosome conformation capture (3C) assay [27], [28], [29], [30]. We selected PstI restriction enzyme since it has cleavage sites throughout the *Col2a1* locus and we chose three cleavage sites near the *Col2a1* promoter (+607), the 3′ UTR (+27298) and between them (+6502) (Fig. 4A). Primer pair A+B and E+F flanked the Pst1 sites, which are closely located to the promoter and 3′ UTR, respectively (Fig. 4A). Primer pair C+D flanked the Pst1 sites located between the promoter and the 3′ UTR (Fig. 4A). Primer pair X+Y is used to amplify the genomic DNA locus that does not contain any Pst1 site (as the loading control). 3C assay samples were prepared as detailed in Materials and Methods section. Primary chondrocytes were treated with formaldehyde to fix their DNA conformation. Then, the cross-linked DNA complexes were digested with the Pst1 restriction enzymes and treated with ligase to join together ends of DNA which were in reasonable physical proximity. Digestion efficiency was confirmed to be more than 95% (P0 stage in Fig. S2A). The ligated products were analyzed by PCR using primer pairs specific for the restriction enzyme containing regions (Fig. 4A). We assessed the formation of 3C product with primer pairs A+D, A+F and F′+A′. We obtained ligated products between A+F, and F′+A′ presumably due to their physical proximity formed by DNA looping (Fig. 4B). Primer pair A+D however generated no significant amount of PCR product since ligated products can be obtained only when the restriction sites are brought close to one another by physical interaction. As a control, we preceded the 3C assay in the absence of cross-linking (data not shown) or ligation (Fig. 4B) and confirmed a significant decrease of 3C product (Fig. 4B). Enriched 3C product with the primer pairs A+F and F′+A′ suggests the close proximity of promoter and 3′ UTR through DNA looping since they are almost 30 kb apart in the chromosomal location. This result suggests that the 3′ UTR physically interacts with the distantly located *Col2a1* promoter to function as an enhancer (Fig. 2C) in primary chondrocyte.

To elucidate the functional role of Lef1 in the gene looping, we tested the effect of knock-down of Lef1 on *Col2a1* expression as well as on gene looping in *Col2a1* locus. Transfection of Lef1 siRNA into primary chondrocytes significantly reduced the amount of 3C product formation by primer pairs A+F or F′+A′ (Fig. 4B and gray bar in Fig. 4C) as well as the *Col2a1* expression (Fig. 3D). This result suggests a pivotal role of Lef1 in DNA looping dependent *Col2a1* expression.

### Decreased Chromatin Accessibility of *Col2a1* Locus during Chondrocyte Dedifferentiation

**Figure 5:**
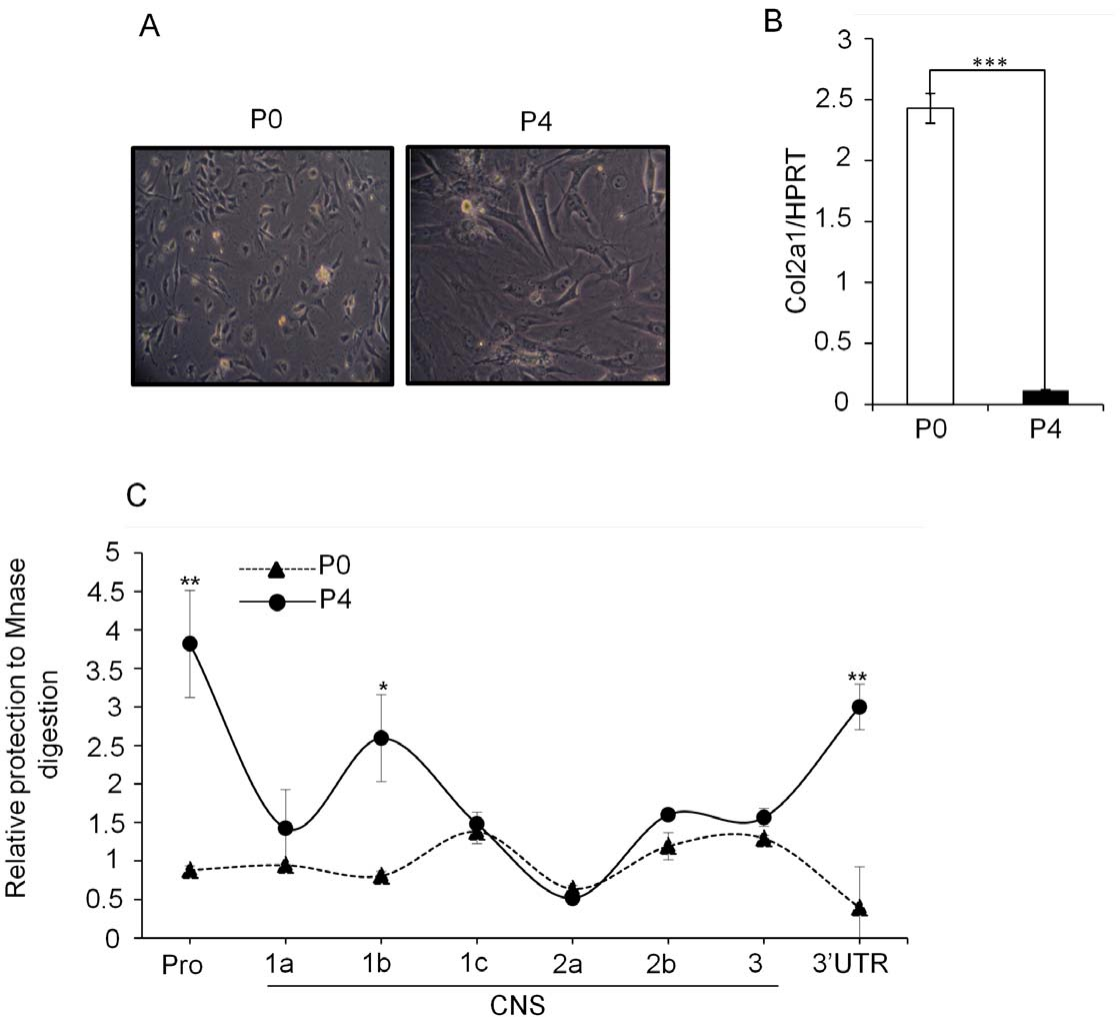
Down-regulation of *Col2a1* expression during chondrocyte dedifferentiation. (A) Primary chondrocytes were isolated from ribs of 4–5 days-old ICR mice and subcultured three more times at 2–3 days intervals to get the terminally differentiated stage (passage P4). Phase contrast images of primary chondrocytes (passage P0) and terminally differentiated passage P4 are represented. (B) Relative level of *Col2a1* transcript in the P0 and P4 stage of chondrocytes was detected by qRT-PCR and expressed relative to the level of housekeeping control HPRT. (C) Chromatin accessibility test by CHART-PCR. Nuclei were prepared from P0 and P4 stage cells treated with MNase and genomic DNAs were isolated. 50 ng of genomic DNA from each stage was used to perform qRT-PCR using primer pairs specific for the promoter, CNSs and the 3′ UTR of *Col2a1* locus. The relative digestion to MNase was calculated with respect to that of actin promoter primers as described in Materials and Methods section. The data shown are expressed as mean ± SEM, n = 3 and *P<0.05, **P<0.01 and ***P<0.001.

Since down-regulation type II collagen expression is associated with cartilage degenerative diseases, we further questioned whether chondrocyte dedifferentiation is associated with any defect in the Lef1 mediated gene looping. We adopted the well accepted chondrocyte dedifferentiation model by sub-culturing chondrocyte for several stages [31]. We first isolated mouse articular chondrocytes (MAC) from 4 days old ICR mouse as described in Materials and Methods section. Cells were plated and cultured until confluent (passage P0). The cells were then dissociated with trypsin and subcultured three more times at 2–3 days intervals to get the terminally differentiated stage (passage P4). Cells from P0 and P4 stages were photographed. As reported previously [7], the morphology of chondrocytes from P4 stage cells were dramatically different from that of P0 stage with a flattened phenotype and much larger in size (Fig. 5A). The level of *Col2a1* transcript also significantly different between the two stages, which indicate that type II collagen expression, is under tight transcriptional regulation (Fig. 5B).

Next, we tested whether down-regulated *Col2a1* expression during chondrocyte dedifferentiation is also associated with differential chromatin accessibility at the regulatory regions of *Col2a1* locus. We assessed the chromatin configuration between P0 and P4 stage by testing the differential chromatin accessibility to MNase treatment. Relative amount of protected product was quantitated by qRT-CR assay with primer pairs for each indicated CNSs as described in Fig. 1B. Nuclei prepared from P0 and P4 stage of cells were left untreated (undigested) or incubated with MNase (digested), and genomic DNAs were isolated as described in the Materials and Methods section. 50 ng of genomic DNA from each stage was used to perform qRT-PCR. Accessibility was calculated relative to the product obtained with the primers for actin promoter and expressed as relative value as described in Materials and Methods section. The amount of product generated is inversely proportional to the amount of digestion. We found that along with the promoter and the first intron (CNS1b; +2014/+2405) [25], the 3′ UTR also became inaccessible to MNase digestion as the chondrocytes undergo dedifferentiation from P0 to P4 stage (Fig. 5C). This result suggests that decreased chromatin accessibility can be accounted as the acquisition of a more compact chromatin structure in the P4 stage, which signifies transcriptionally inactive state marked by decreased production of *Col2a1* transcript.

### Permissive *Col2a1* Chromatin Structure in the Primary Chondrocytes and Preferential Binding of Lef1 to the 3′ UTR

**Figure 6:**
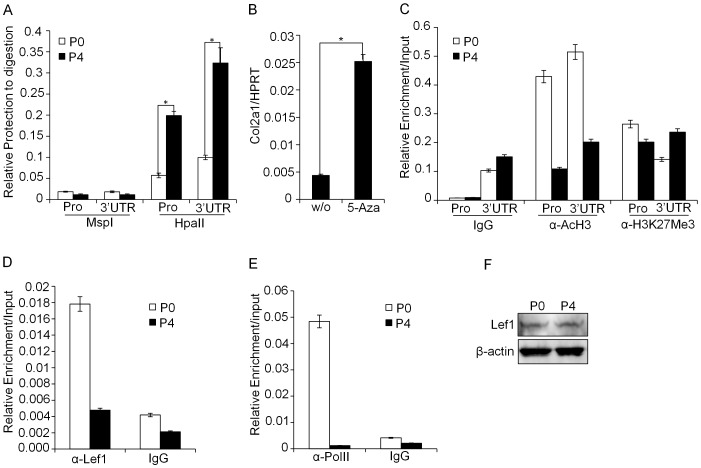
Epigenetic modifications at the regulatory regions regulate *Col2a1* expression levels. (A) Analysis of methylation sensitive restriction profile on the Col2a1 promoter and 3′ UTR was performed by digesting nuclei from P0 and P4 stage cells. After digestion with HpaII and its isochizomer MspI, followed by genomic DNA isolation, 50ng of each sample was used to perform qRT-PCR using primer pairs specific for the promoter and the 3′ UTR of *Col2a1* gene. (B) P4 stage cells were left without treatment or treated with methylation inhibitor 5-Azacytidine (5-Aza) and relative level of Col2a1 transcript was compared by qRT-PCR. All the data are representative of three independent experiments. (C) ChIP assay was performed with crosslinked chromatin from P0 and P4 stage of chondrocytes using antibodies such as AcH3 (active marker), H3K27Me3 (inactive marker) and rabbit IgG. The enriched target DNA level in each of the precipitated samples was assessed by qRT-PCR and plotted. (D, E) Physiological binding of Lef1 (D) or Pol II (E) to the 3′ UTR or promoter, respectively was assessed by ChIP assay. Crosslinked and fragmented DNA from P0 and P4 stage chondrocytes were immunoprecipitated with Lef1 antibody, anti-Pol II antibody or rabbit IgG (as a control). The enriched target DNA level was assessed by qRT-PCR. (F) The levels of Lef1 protein expression in the P0 and P4 stage chondrocytes were analyzed by immunoblotting with α-Lef1 and α-β-actin (control) antibodies. The data shown are expressed as mean ± SEM, n = 3 and *P<0.05.

The transcriptional effects of regulatory elements are often associated with epigenetic changes such as histone modifications and DNA methylation status. We tested whether the epigenetic modification at the promoter and 3′ UTR contributes to the stage specific differential expression of *Col2a1*. First, we analyzed differential DNA methylation status between P0 and P4 stage chondrocytes by using methyl sensitive restriction enzyme HpaII and its isochizomer MspI. When methylation occurs at the second C in the CCGG target sequence, HpaII is not able to recognize the target site, however, MspI can recognize the target site regardless of methylation status. Similar to CHART-PCR analysis, the amount of product obtained is inversely proportional to the amount of digestion. We found CCGG sites both in the *Col2a1* promoter and 3′ UTR and tested their methylation status. Same amount of HpaII/MspI digested DNA from the P0 and P4 stages were subjected to qRT-PCR using primers specific for the promoter and 3′ UTR. Indeed, both the promoter and 3′ UTR of *Col2a1* locus showed a high resistance to HpaII digestion in the P4 stage; however, P0 stage showed high susceptibility to both enzymes (Fig. 6A). To further validate this result, we tested the effect of 5-Aza (5-Aza-2′-deoxycytidine; an inhibitor of DNA methylation) treatment on *Col2a1* expression. Indeed, 5-Aza treatment significantly increased *Col2a1* expression in P4 stage chondrocytes, although its expression level was still much lower than the P0 stage cells (Fig. 6B and data not shown). Next, we compared the level of recruited transcriptionally active and inactive marker histones onto the promoter and 3′ UTR. ChIP analysis was performed with the chromatins prepared from P0 and P4 stage of chondrocytes. Compared with P0 chondrocyte, a significant decrease of AcH3 (Acetylated histone H3) recruitment, a marker of active chromatin, to the *Col2a*1 promoter and 3′ UTR was observed in P4 stage of chondrocyte. However, no difference was detected in the levels of inactive histone marker H3K27Me3 (Histone 3 Lysine 27 trimethylation) between the two stages (Fig. 6C).

Finally we tested the functional relationship between the accessibility of chromatin architecture and recruitment of Lef1 to the 3′ UTR and its effect on *Col2a1* expression. *In vivo* Lef1 binding to the 3′ UTR was compared between primary chondrocyte (P0 stages; high expression of *Col2a1*) and P4 stages of dedifferentiated chondrocyte (low expression of *Col2a1*) by ChIP assay. As expected, much higher level of Lef1 binding was observed in P0 stage compared with P4 stage of chondrocyte (Fig. 6D). In addition, enrichment of RNA Pol II to the Col2a1 promoter in the P0 stage was also significantly higher than that of P4 stage of chondrocyte (Fig. 6E). However, Lef1 protein levels were similar between the P0 and P4 stages as determined by immunoblotting (Fig. 6F). Taken together, these results demonstrate that epigenetically permissive chromatin architecture in the *Col2a1* regulatory loci allows recruitment of architectural transcription factor Lef1, which leads to enhance *Col2a1* expression through gene looping.

## Discussion

Homeostasis of type II collagen levels in articular cartilage is important for providing tensile strength to the cartilage tissue and its disrupted expression is associated with bone and cartilage degenerative diseases. In this study, we have identified a novel function of 3′ UTR as a *cis*-acting DNA element in regulating *Col2a1* transcription in chondrocytes. Recruitment of Lef1 transcription factor to the 3′ UTR of *Col2a1* locus induced a DNA loop, which brings the promoter and the 3′ UTR to close proximity resulting in up-regulation of *Col2a1* gene transcription. However, acquisition of a compact chromatin structure in the dedifferentiated stage in chondrocytes leads to the decreased recruitment of Lef1 to the 3′ UTR, which subsequently results in significant reduction of type II collagen expression.

Identification of *cis-* and *trans-*acting elements involved in transcriptional regulation of *Col2a1* gene expression might be challenging since its genomic locus comprises around 50 kb. Indeed, only the first intron so far has been identified as an enhancer [8], [20], [21], [22], [23]. In this study, we performed bioinformatics analysis on genomic locus of mouse and human *Col2a1* and identified a novel function of 3′ UTR as a potent enhancer. Although 3′ UTR alone has basal promoter activity, it showed potent enhancer activity upon coupling with *Col2a1* promoter (Fig. 1). Lef1 binding to the 3′ UTR is crucial to up regulate *Col2a1* transcription (Fig. 3). Knock-down of Lef1 expression by Lef1 siRNA in primary P0 stage chondrocytes significantly decreased the transactivity of the 3′ UTR (Fig. 2C and Fig. 3A–D). However, in reporter assay, mutation of Lef1 binding site in the 3′ UTR failed to decrease its enhancer activity (data not shown). These results suggest that although Lef1 plays a pivotal role, other TCF/Lef family transcription factors may be also involve in upregulation of 3′ UTR enhancer activity by forming a transcriptome complex at the 3′ UTR-promoter region. TCF7 (TCF-1), TCF7L1 (TCF-3), TCF7L2 (TCF-4) and Lef-1 are group of TCF/Lef family transcription factors which bind to DNA [32] through a high mobility group domain [33], [34]. Expression of TCF-3, TCF-4 and Lef1 have been reported in the cartilage [35]. Upregulated TCF-1 expression is confined to the prehypertrophic chondrocytes and in the surrounding perichondrium. TCF-3 can be detected in the whole cartilage, while hypertrophic chondrocytes in Col2a1-ICAT transgenic mice only express TCF-4 [36], [37], [38], [39]. Thus, different Lef/TCF family members have differential expression pattern in the different stages of chondrocyte maturation. However, further studies are needed to clarify the issue whether other Lef1-related proteins could also bind to the 3′ UTR thereby leads to upregulate *Col2* gene expression through gene looping.

Lef1 introduces a sharp bending of about 130 degrees in the DNA and loop the DNA bringing distantly parted *cis*-element to close proximity [27]. Gene looping is a well-established method by which distant *cis*-elements coordinately regulate the transcription of specific genes [28]. Formation of a gene loop stabilizes the active transcriptional machinery and triggers new rounds of transcription and this results in an accelerated and efficient transcription [29]. In this study, we found that over-expression of Lef1 increased *Col2a1* expression while knock-down of Lef1 exerted opposite effect (Fig. 3A–D). These findings prompted us to ask how does the binding of Lef1 to the distal 3′ downstream region of *Col2a1* locus regulate *Col2a1* gene transcription? Previously we have reported that Lef1 binding to the 3′ UTR of *Cox2* and *MMP13* genes can regulate their transcriptional activity through gene looping [30]. Therefore we tested whether Lef1 mediated enhancer activity of 3′ UTR is associated with gene looping between the 5′ located promoter and 3′ located UTR region of *Col2a1*. Indeed, 3C analysis showed that *Col2a1* transcription is regulated by Lef1 dependent gene looping. Knockdown of endogenous Lef1 level in primary chondrocytes significantly decreased the incidences of gene looping (Fig. 4). Hence recruitment of Lef1 in the extreme 3′ end of the gene is a mechanism to facilitate active *Col2a1* transcription. Lef1 is involved in Wnt signaling where in combination with β-catenin it up regulates the expression of several genes [30], [40], [41], [42], [43], [44]. Although Wnt signaling is associated with the progression of arthritis, several reports also showed that impairment of β-catenin signaling can lead to arthritis and is responsible for maintenance of healthy cartilage [35]. β-catenin, the coactivator of Lef1, probably has a dual role in the proper maintenance of healthy cartilage by balancing the anabolic and catabolic processes. It has been demonstrated that β-catenin is essential for the early stages of cartilage growth and development whereas over-expression of β-catenin in the adult cartilage is accompanied by cartilage destruction [45]. Inhibition of β-catenin signaling in chondrocytes resulted in significant reduction of expression of Lef/TCF family members such as Lef1, TCF-3 and TCF-4 and showed defects in post-natal cartilage development [35], [46] and delayed chondrogenesis which is accompanied by significant decrease in the cartilage marker gene *Col2a1* [46], [47]. Absence of Lef1 leads to increased apoptosis of chondrocytes [35] and similar effect is also found upon Col2a1 deficiency [48]. Lef1 suppressed cells reduced expression of *Col11a1* [49], [50]. Moreover, over-expression of a dominant negative form of Lef1 results in the inhibition of chondrogenic differentiation by decreasing the expression of *Col2a1* and aggrecan genes [41].Thus our study also supports the above result and suggests the role of Lef1 as a positive regulator for *Col2a1* expression during the normal process of chondrocyte differentiation. High level expression of β-catenin may contribute cartilage growth in the early stage of development. However, adult healthy cartilage expresses low levels of β-catenin, while over-expression of β-catenin is associated with cartilage destruction [45]. Like the β-catenin, Lef1 may also exhibit a dual function depending upon the physiological state of the chondrocytes. Further investigations are required to elucidate the exact role of Lef1 in chondrocyte differentiation. Beside, Wnt independent role of Lef1 is also reported where it is shown that the β-catenin independent DNA binding domain of Lef1 can transactivate the Col11a1 promoter and Lef1 suppressed cells have reduced expression of *Col11a1* [49], [50]. Interestingly, *Col11a1*, a heterotrimer of alpha 1 (XI) collagen (Col11a1), alpha 2 (XI) collagen (Col11a2) and alpha 1 (II) collagen, is mainly expressed in the articular cartilage and vitreous fluid of the eye, and is responsible for the proper type II collagen fibril formation [51], [52]. Mutation of Type XI collagen resulted in accumulation of degraded type II collagen in articular cartilage [53]. These findings suggest a physiological relevance of the β-catenin-independent function of Lef1 in maintaining healthy cartilage. In addition, removal of the β-catenin interacting N-terminal domains from Lef/Tcf proteins resulted in increase of DNA bending-mediated gene regulation activity of Lef/Tcf family [54]. These studies indicate that members of Lef/Tcf family can activate gene expression independent of β-catenin [58]. However, future studies are necessary to clarify the role of Wnt signaling in Lef1-mediated *Col2a1* expression.

Dedifferentiation of chondrocyte is associated with significant decrease of type II collagen [1], [55], [56]. This phenomenon occurs in the arthritic processes and can be mimicked *in vitro* by sub-culturing monolayer cultures of chondrocytes from both mouse and human [7], [55], [57]. We tested whether Lef1 mediated *Col2a1* regulation is also associated with dedifferentiation of chondrocytes by adapting the *in vitro* chondrocyte differentiation model. Compared with primary chondrocyte (P0 stage), dedifferentiated chondrocyte (P4 stage) expressed significantly lower *Col2a1* transcript (Fig. 5A–B). Analysis of chromatin accessibility and epigenetic statues revealed that P4 stage of chondrocyte attains a compact and condensed structure (Fig. 5C and Fig. 6A–C), which makes it inaccessible to the transcription factor machinery. This finding was well correlated with the decreased binding of Lef1 to the 3′ UTR in the P4 stage and consequent decrease of Pol II binding to the promoter (Fig. 6D and E). All these events indicated that 3′ UTR of Col2a1 in conjunction with the promoter plays regulatory role in Col2a1 expression by epigenetic modifications.

In conclusion, we identified the 3′ UTR as a novel enhancer for *Col2a1* expression. Recruitment of Lef1 to the 3′ UTR is crucial for *Col2a1* up regulation by inducing gene looping under the permissive chromatin architecture of primary chondrocyte. However, it is still possible that unidentified CNSs and their interacting transcription factors have additional roles to regulate *Col2a1* transcription. Systemic elucidation of functional crosstalk between the *cis*- and *trans*-acting elements involved in *Col2a1* regulation can lead to development of therapeutic interventions for arthritis and other cartilage degenerative diseases.

## Materials and Methods

### Animals and Cell Line

ICR (Imprinting control region) mice were housed in specific pathogen-free barrier facilities and used in accordance with protocols approved by the Animal Care and Ethics Committees of the Gwangju Institute of Science and Technology (GIST). The human chondrosarcoma cell line, HTB-94 (ATCC, Manassas, VA) [58] was maintained in Dulbecco′s modified Eagle′s medium (DMEM) supplemented with 10% fetal bovine serum (Hyclone Laboratories, Logan, USA), 100 U/ml penicillin (Sigma-Aldrich; St. Louis, MO) and 100 g/ml streptomycin (Sigma-Aldrich; St. Louis, MO) in a humidified 37°C incubator and was subcultured at regular intervals.

### Culture of Mouse Rib Chondrocytes

Primary chondrocytes were isolated from ribs of 4-day-old ICR mice and cultured as previously described [42], [59], [60]. The chondrocytes released from cartilage were suspended in DMEM supplemented with FBS (10% (v/v); Hyclone Laboratories, Logan, USA), streptomycin (50 µg/ml), and penicillin (50 U/ml) and then plated on culture dishes at 2−4×10^4^ cells/cm^2^ and cultured until confluence (passage P0). The cells were then dissociated with trypsin-EDTA and subcultured three more times at 2–3 days intervals to get the terminally differentiated stage (passage P4).

### RNA Isolation, cDNA Synthesis, Quantitative Real-time PCR (qRT-PCR)

Total RNA was extracted from the P0 and P4 stage of chondrocytes using TRIZOL Reagent (Molecular Research Center; Cincinnati, OH) according to the manufacturer’s protocol. For reverse transcription, 1 µg of total RNA was used and cDNA was generated using oligo (dT) primer (Promega; Madison, WI) and Improm-II Reverse Transcriptase (Promega; Madison, WI) in a total volume of 20 µl. The mRNA level was determined using 1 µl of cDNA by real-time PCR with SYBR green using the protocol provided by the manufacturer (MJ Research Chromo4). The primer sequences used are as follows: Col2a1 (F-5′- TCG CAC TTG CCA AGA CCT GAA A -3′ and R-5′- TTT CCT TGC TCT TGC TGC TCC A-3′). Mouse hypoxanthine-guanine phosphoribosyl transferase (HPRT) primer HPRT (F-5′-TTA TGG ACA GGA CTG AAA GAC-3′ and R-5′-GCT TTA ATG TAA TCC AGC AGG T-3′), was used for quantitative RT-PCR to normalize the amount of cDNA used for each condition.

### Computational Analysis of the *Col2a1* Locus

To identify conserved non-coding sequences (CNSs) as potential regulatory elements, mouse and human genomic sequence spanning the 30 kb of the *Col2a1* gene was analyzed using the web-based alignment software, VISTA browser 2.0. Transcription factor binding sites were identified using the rVISTA program [61], which uses matrices of the TRANSFAC database [62]. The positions of the analysed CNSs with respect to the transcription start site (+1) are indicated in the Figure S1.

### Chromatin Accessibility Assay

Chromatin accessibility by Real Time PCR (CHART-PCR) was performed as described previously [63], [64] by combining MNase accessibility assay with qRT-PCR. Briefly, P0 and P4 stage cells were pelleted by centrifugation at 1500 rpm at 4°C. Cells were washed twice in cold PBS and resuspended in NP-40 Lysis buffer (10 mM Tris [pH 7.4], 10 mM NaCl, 3 mM MgCl_2_, 0.5% NP-40, 0.15 mM spermine, 0.5 mM spermidine) [65] and incubated in ice for 10 min, centrifuged and then resuspended in 100 µl digestion buffer (10 mM Tris-HCl (pH 7.4), 15 mM NaCl, 60 mM KCl, 0.15 mM spermine, 0.5 mM spermidine, 1 mM CaCl_2_) with or without 5 U MNase/ml (Roche; Mannheim, Germany) and were further incubated at 37°C for 10 min. Reactions were terminated by adding 20 µl stop solution (100 mM EDTA, 10 mM EGTA - pH 8.1) and 10 ml SDS 10% (w/v). DNA was isolated using the DNA blood genomic prep kit (Intron Biotechnology; Daejon, Korea) and eluted into 100 µl TE. DNA recovered from MNase samples was checked for fragmentation in 1% agarose gel. Isolated genomic DNA from each samples were quantified spectrophotometrically and 50 nanograms of each sample were subjected to SYBR green based qRT-PCR to measure the relative abundance of target regions using the primer sets described in Table 1. Each reaction was done in duplicate. Primers for actin promoter were used as a normalization control to estimate product formation. Chromatin accessiblity values were calculated as a ratio of the product formation in the digested samples to that of the undigested samples and plotted. ΔCt values were calculated as Ct (cut-uncut) for each primer pairs against the respective regions including those of actin promoter. ΔC(t) values of each samples were then normalised to the ΔC(t) value of actin promer and expressed as relative accessibility and plotted.

**Table 1:** Primer sequences for CHART-PCR and ChIP assay.

Primer position	Sequences (5′ - 3′)	CNS
−57/+166	F : GTTTGCCAGCCTTTGGAGC	Pro
	R : CGAGGCGGATCATGGCTCAC	
+976/+1178	F : CTATCGTTAGAGGTGGCAGCTGTATAG	CNS 1a
	R : CATACAGAGAAAGACAGGGCTGGAAC	
+2014/+2405	F : GACATTGACCCACATCTGCATTTCTCAG	CNS 1b
	R : GCTACCTCTTTCGGGGAACTGTTTTG	
+2900/+3068	F : CTGAGGTGGAGGAGCAGGGAGTATC	CNS 1c
	R : TCTGCTCTTCAAGGAGGGCGAGAATCC	
+6397/+6663	F: GCTTCTAAATTGCTACTCTCTTACCTGGCAGC	CNS 2a
	R: CCAGAGAAATACAAGTGCCCAGTCATTCTTTG	
+7628/+7767	F : CTGTTCTTCAGCATCCACCAGGCTTC	CNS 2b
	R : CATTTGGACCGAGACGCTGGCCTTG	
+14049/+14335	F : GTGGGCAAAAGCTGAGGTACCCAGAAGG	CNS 3
	R : CAGGCGTAGAAAAGCAACCTGATGCCAG	
+28700/+29122	F : CTGACCTGACCTGATGATACCCA	3′ UTR
	R : GGGCATGCCTTATAGAACCAAGG	

Region encompassed by the analyzed CNSs are:

Promoter;−57/+166, CNS 1; +850/+3334, CNS 2; +5755/+9165, CNS 3; +14013/+14613, 3′ UTR; +28700/+29243.

### Plasmid Construction, Site Directed Mutagenesis and Luciferase Reporter Assays

The Col2a1 minimal promoter (−57/+166) and the 3′ UTR constructs (full length 543 bp: +28700/+29243; 189 bp: +29054/+29243; 134 bp: +29110/+29243) were cloned in the luciferase reporter vector pXPG and were confirmed by sequencing. HTB-94 human chondrosarcoma cells and P0 stage of mouse chondrocytes were transfected with the reporter constructs and or with plasmid encoding Lef1 using METAFECTENE (Biontex; Luzern, Switzerland) according to the manufacturer’s protocol. After 18–24 hrs, luciferase activity was measured by the dual luciferase assay system (Promega; Madison, WI). pRLTK was cotransfected with each sample to normalize the data by the activity of Renilla luciferase.

### Chromatin Immunoprecipitation (ChIP) Assays

ChIP analysis was carried out essentially as described [42] with minor modifications. P0 and P4 stage chondrocytes were cross-linked with formaldehyde (Sigma-Aldrich, St. Louis, MO) and DNA was fragmented by sonication to obtain 500–1000 bp fragment sizes on average. Antibodies against Lef1 (polyclonal SC-8591, Santa Cruz Biotechnology Inc., Santa Cruz, CA), RNA Pol II (Santa Cruz Biotechnology Inc., Santa Cruz, CA), acetyl histone H3 lysine9/14 (H3AcK9/14; Upstate; Lake Placid, NY), trimethyl histone H3 lysine 27 (H3K27Me3) (Upstate; Lake Placid, NY) and rabbit IgG (Sigma; St. Louis, MO) were used to immunoprecipitate specific DNA regions. Following reverse cross-linking the presence of target DNA sequences was assessed by RT-PCR. The primers used are indicated in Table 1. Control or input DNA was obtained before immunoprecipitation and the amount of product formation was interpreted relative to PCR product obtained from the input DNA.

### Immunoblotting

Chondrocytes (P0, P4 stage, Lef1-transfected P0 stage, Lef1 siRNA treated P0 stage) were lysed in cell lysis buffer (50 mM Tris, pH 8.0, 0.5% Nonidet P-40, 10% glycerol, 0.1 mM EDTA, 100 mM NaCl, 50 mM NaF, 1 mM Na3VO4, 1 mM Dithiothreitol (DTT), protease inhibitor cocktail (Roche; Mannheim, Germany)) and protein concentrations were estimated. Thirty micrograms of whole cell lysate was used for SDS-PAGE and Western blot was carried out with α-Lef1 antibody. The same membrane was stripped with mild-stripping buffer (Abcam; Cambridge, MA) and reprobed with α-β-actin (Abcam; Cambridge, MA) antibodies.

### Nuclear Extracts and Electrophoretic Mobility Shift Assay (EMSA)

To prepare nuclear extract, P0 stage chondrocytes were washed in ice cold PBS and suspended in 1ml of lysis buffer (10 mM Tris/HCl, 3 mM CaCl_2_, 2 mM MgCl_2_) containing protease inhibitor cocktail (Roche; Mannheim, Germany) for 10 min on ice and centrifuged at 3000 rpm for 10 min at 4°C. The pellet obtained were incubated in 1 ml of NP-40 buffer (10 mM Tris/HCl, 3 mM CaCl_2_, 2 mM MgCl_2_, 1% NP-40) for 5 min at 4°C and centrifuged at 3000 rpm for 10 min at 4°C. Nuclei were washed with 1 ml of Buffer A (20 mM HEPES-KOH, 1.5 mM MgCl2, 10 mM KCl, 0.5 mM DTT, 0.5 mM PMSF). Nuclei were lysed with 100 µl of Buffer C (20 mM HEPES-KOH, 25% Glycerol, 420 mM NaCl, 1.5 mM MgCl_2_, 0.2 mM EDTA, 5 mM DTT, 0.5 mM PMSF, 1% Triton X by vortexing vigorously at 4°C for 10 min and protein concentration was estimated. For the EMSA probes the forward primer sequences are as follows: WT (corresponding to the Lef1 biding site in the 3′ UTR ) 5′- CTT GTG TTT TGT TCT TTG TTT TG-3′ and Lef1 consensus: 5′AAT TCC GGC CTT TGA TCT TTG CTA-3′ [26]. Complementary oligonucleotide pairs were annealed in 100 mM NaCl, 10 mM Tris pH 8.0, 0.1 mM EDTA by heating to 95°C for 10 min, and slow cooling. [γ-^32^P] ATP (PerkinElmer; Waltham, MA) and T4 polynucleotide kinase (Promega; Madison, WI) were used to end label the double-stranded oligonucleotides which were subsequently purified using G-50 column (275330, Amersham Phamacia Biotech; Piscataway, NJ). Labeled probes were incubated with prepared nuclear extract (2 µg) in binding buffer (10 mM Tris pH 7.5, 0.5 mM MgCl_2_, 80 mM NaCl, 2.5 mM DTT, 4% glycerol, 1 mM-mercaptoethanol, 20 ml) along with 0.1 mg/ml poly (dI-dC) at 4°C for 30 minutes. For cold competition, unlabeled wild type, probe or unlabeled consensus was added and preincubated for 20 min. For super shift assays, nuclear extracts were preincubated for 30 min with 2 µg of anti-Lef1 antibody (Santa Cruz; Santa-Cruz, CA). The samples were separated in 4% nondenaturing polyacrylamide gel containing 0.5× Tris-borate-EDTA at 4°C.

### Methyl Sensitive Restriction Enzyme Assay

Methyl-sensitive PCR was performed using HpaII/MspI enzyme pair to analyze the methylation status of the Col2a1 promoter and 3′ UTR. Genomic DNA was prepared from P0 and P4 stage using a blood DNA extraction kit (Qiagen; Duesseldorf, Germany) and 1 µg was digested with 10 U of HpaII or MspI enzymes overnight at 37°C. The target regions were subjected to by RT-PCR with SYBR green using a protocol provided by the manufacturer (MJ Research Chromo4) with primers provided in Table1. Product formation was calculated relative to that of uncut DNA from each stage.

### 5-aza-2′-deoxycytidine Treatment

Chondrocytes at P4 stages were cultured in medium containing with 5 µM of 5-aza-2′-deoxycytidine (5-Aza; Sigma-Aldrich; St. Louis, MO) that was freshly diluted immediately before use, for 3 days by replacing the media with fresh 5-Aza containing media every 24 hrs.

### Lef1 Over-expression and Lef1 Specific Small Interfering RNA (siRNA) Transfection

Chondrocytes at the P0 stage were transfected with plasmids encoding Lef1, Lef1 siRNA and control siRNA and (SC-35805, Santa Cruz Biotechnology Inc., Santa Cruz, CA) using Lipofectamine Plus (Invitrogen; Carlsbad, CA), and cultured in complete medium for 48 hrs and total RNA was isolated as described previously.

### Chromosome Conformation Capture (3C) Assay

To detect interaction between distal 3′ UTR with promoter of Col2a1, 3C assay was performed as described previously with minor modifications [66]. Briefly, nuclei prepared from uncrosslinked and formaldehyde crosslinked P0 and P4 stage chondrocytes were subjected to Pst1 digestion. Digestion efficiency was confirmed by RT-PCR using primers flanking the Pst1 (NEB; Ipswich, MA) sites and above 95% digested chromatins were ligated by T4 DNA Ligase (NEB; Ipswich, MA). The formation of 3C product was detected by RT-PCR using digested (cut) and ligated (cut+ligated) DNA samples as templates. PCR products were resolved on 2% agarose gels or cloned into pGEM-T easy vector and sequenced. Quantitative levels of 3C assay product was analyzed by qRT- PCR and normalized by input control generated with primer pair X+Y to amplify the region that does not containing Pst1. The primers used for 3C assay are shown in Table 2.

**Table 2:** Primer sequences for Chromosome Conformation capture Assay.

Name Sequences (5′ - 3′)	Position
A CACAGACGCATCACCTTCCACCAGC	+471
B CAAGGGGAGAGCCGAGTTTCAAAG	+768
C CTGTTCTTCAGCATCCACCAGGCTTC	+6398
D CATTTGGACCGAGACGCTGGCC	+6632
E GGTGAAGGACCATGACAGAAGTGAC	+27085
F GCAAGTCTCGCCGGTCTCCATGTTGCAG	+27360
A′ GTGAATTCCTACACAGAGGGAG	+30808
F′ CGGCTGACTTCACATCTAACC	−679
X ACATTAGCTTCTCTGACTCAC	+16801
Y CTCACCCAAACTCCCTTCTC	+17455

### Statistical Analysis

Data are the mean of SE of at least three independent experiments, unless differently specified in the text. The student’s *t*-test was used to determine the significance of the results. The level of significance was set at *P<0.05, **P<0.01 and ***P<0.001. Significance was only indicated when appropriate.

## Supporting Information

Figure S1 **Comparative sequence analysis of the mouse and human** ***Col2a1*** **loci.** Comparison of the genomic sequence of murine and human 3′ UTR region. Positions are assigned with respect to the transcription start site (TSS). Potential binding sites for Lef1 that met the most stringent requirements (matrix similarity values (0.8), using rVISTA 2.0 and TRANSFAC database analysis, are identified and boxed. Primer pair at position +29054 (indicated by bold letter C) and +29243 generate a 189 bp 3′ UTR construct and primer pair at position +29110 (indicated by bold letter A) and +29243 generate a 133 bp 3′ UTR construct.
(PDF)

Figure S2 **Differential chromatin accessibility between P0 and P4 stage of chondrocytes.** Chromatins prepared from non-crosslinked or formaldehyde (HCHO) crosslinked P0 and P4 stage chondrocytes were treated with *Pst1* and digestion efficiency was calculated relative to the product obtained by non-*Pst1* flanking primer pairs X+Y (Fig. 4). Data are expressed as percentage of the product obtained in uncut samples.
(PDF)
