# Supplementary material for: Looping Mediated Interaction between the Promoter and 3′ UTR Regulates Type II Collagen Expression in Chondrocytes
Source: PLoS One. 2012 Jul 16;7(7):e40828. doi: 10.1371/journal.pone.0040828 (PMC3397959; doi:10.1371/journal.pone.0040828)
Supplement: Figure S1 — Comparative sequence analysis of the mouse and human Col2a1 loci. Comparison of the genomic sequence of murine and human 3′ UTR region. Positions are assigned with respect to the transcription start site (TSS). Potential binding sites for Lef1 that met the most stringent requirements (matrix similarity values (0.8), using rVISTA 2.0 and TRANSFAC database analysis, are identified and boxed. Primer pair at position +29054 (indicated by bold letter C) and +29243 generate a 189 bp 3′ UTR construct and primer pair at position +29110 (indicated by bold letter A) and +29243 generate a 133 bp 3′ UTR construct. (PDF) [file pone.0040828.s001.pdf]

Figure S1.

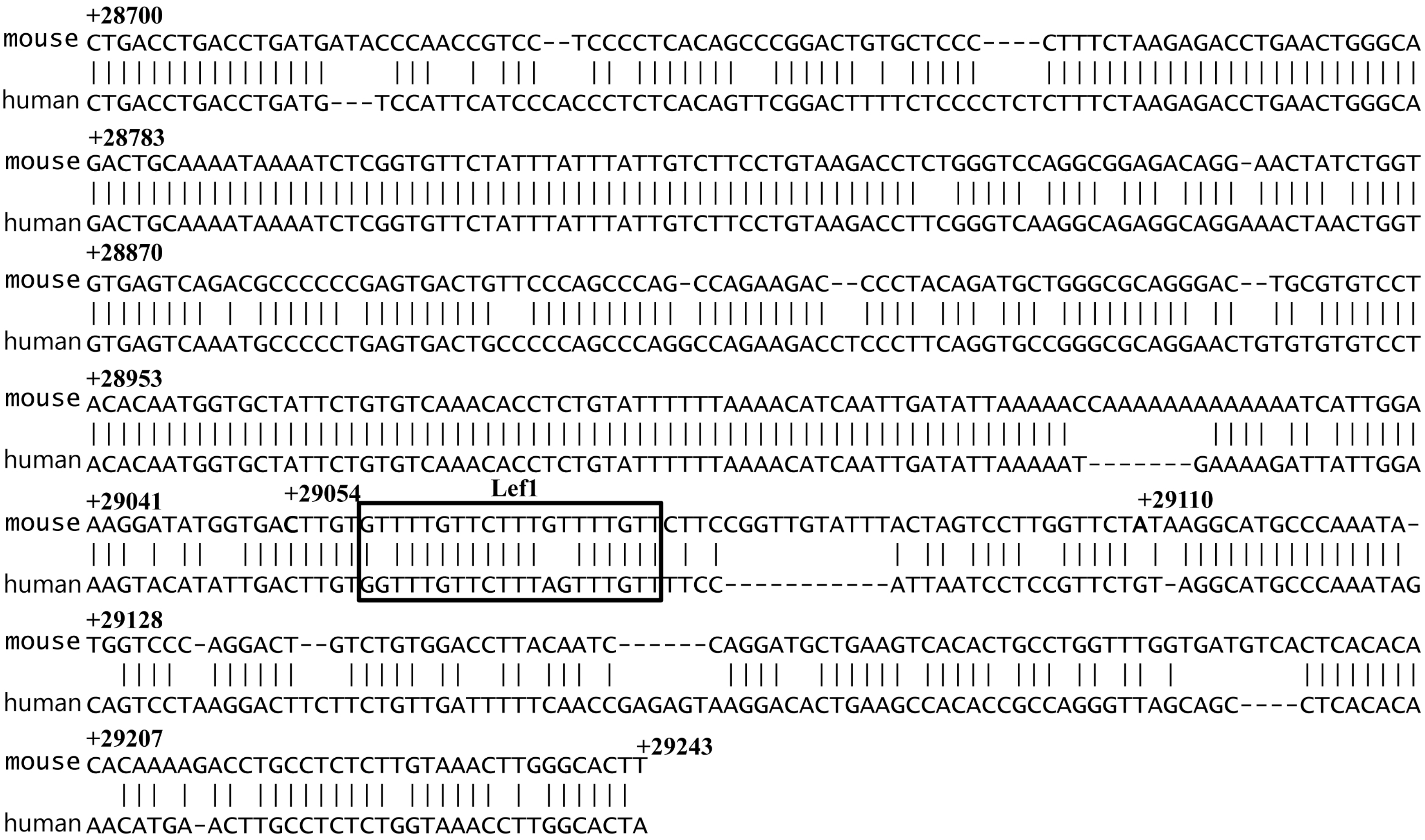
+28700
mouse CTGACCTGACCTGATGATACCCAACCGTCC--TCCCCTCACAGCCCGGACTGTGCTCCC----CTTTCTAAGAGACCTGAACTGGGCA
human CTGACCTGACCTGATG---TCCATTCATCCCACCCTCTCACAGTTCGGACTTTTCTCCCCTCTCTTTCTAAGAGACCTGAACTGGGCA
+28783
mouse GACTGCAAAATAAAATCTCGGTGTTCTATTTATTTATTGTCTTCCTGTAAGACCTCTGGGTCCAGGCGGAGACAGG-AACTATCTGGT
human GACTGCAAAATAAAATCTCGGTGTTCTATTTATTTATTGTCTTCCTGTAAGACCTTCGGGTCAAGGCAGAGGCAGGAAACTAACTGGT
+28870
mouse GTGAGTCAGACGCCCCCGAGTGACTGTTCCCAGCCCAG-CCAGAAGAC--CCCTACAGATGCTGGGCGCAGGGAC--TGCGTGTCCT
human GTGAGTCAAATGCCCCCTGAGTGACTGCCCCCAGCCCAGGCCAGAAGACCTCCCTTCAGGTGCCGGGCGCAGGAACTGTGTGTGTCCT
+28953
mouse ACACAATGGTGCTATTCTGTGTCAAACACCTCTGTATTTTTAAAACATCAATTGATATTAAAAACCAAAAAAAAAAAAATCATTGGA
human ACACAATGGTGCTATTCTGTGTCAAACACCTCTGTATTTTTAAAACATCAATTGATATTAAAAT-------GAAAAGATTATTGGA
+29041
+29054
Lef1
+29110
mouse AAGGATATGGTGACTTGTGTTTTGTTCTTTGTTTTGTTCTTCCGGTTGTATTTACTAGTCCTTGGTTCTATAAGGCATGCCCAAATA-
human AAGTACATATTGACTTGTGGTTTGTTCTTTAGTTTGTTTTCC-----------ATTAATCCTCCGTTCTGT-AGGCATGCCCAAATAG
+29128
mouse TGGTCCC-AGGACT--GTCTGTGGACCTTACAATC------CAGGATGCTGAAGTCACACTGCCTGGTTTGGTGATGTCACTCACACA
human CAGTCCTAAGGACTTCTTCTGTTGATTTTTCAACCGAGAGTAAGGACACTGAAGCCACACCGCCAGGGTTAGCAGC----CTCACACA
+29207
+29243
mouse CACAAAAGACCTGCCTCTCTTGTAAACTTGGGCACTT
human AACATGA-ACTTGCCTCTCTGGTAAACCTTGGCACTA
