# Supplementary figures and images for: Looping Mediated Interaction between the Promoter and 3′ UTR Regulates Type II Collagen Expression in Chondrocytes
Source: PLoS One. 2012 Jul 16;7(7):e40828. doi: 10.1371/journal.pone.0040828 (PMC3397959; doi:10.1371/journal.pone.0040828)

Figure S2.

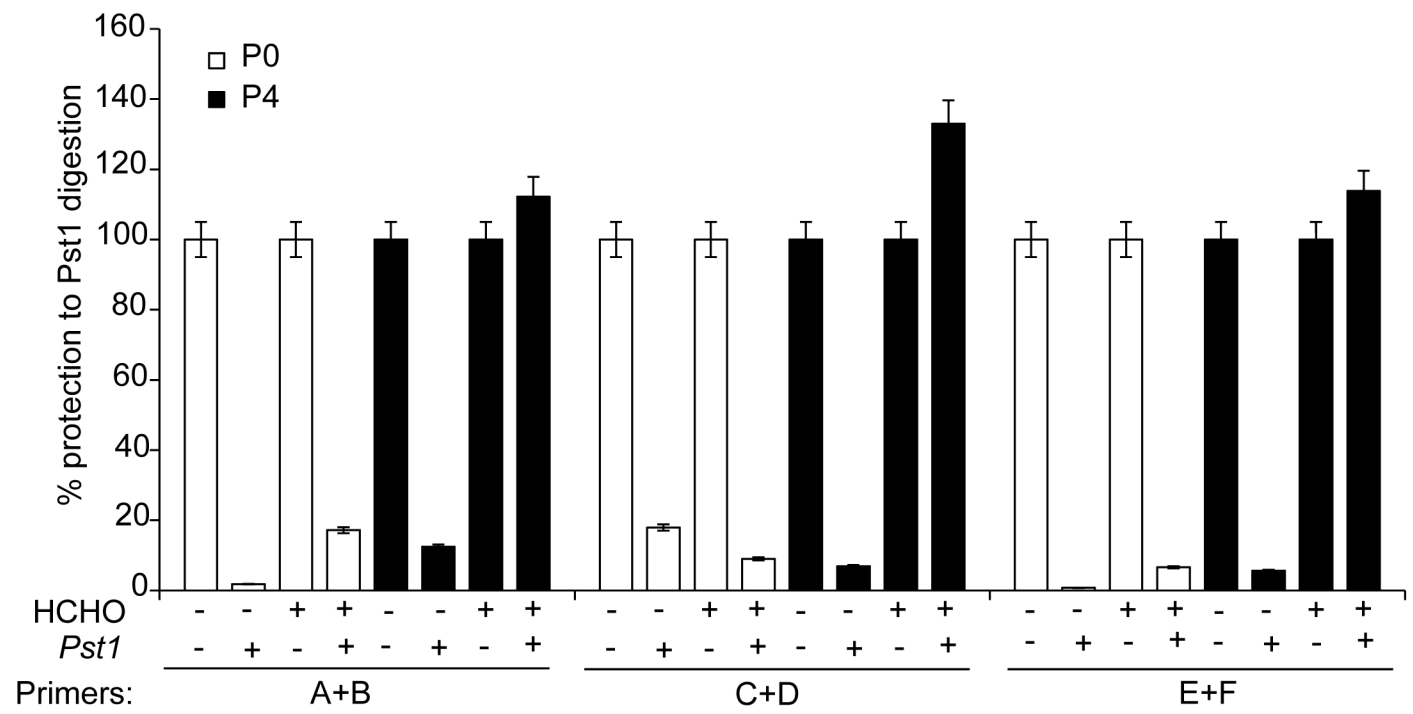

Supplement: Figure S2 — Differential chromatin accessibility between P0 and P4 stage of chondrocytes. Chromatins prepared from non-crosslinked or formaldehyde (HCHO) crosslinked P0 and P4 stage chondrocytes were treated with Pst1 and digestion efficiency was calculated relative to the product obtained by non-Pst1 flanking primer pairs X+Y (Fig. 4). Data are expressed as percentage of the product obtained in uncut samples. (PDF) [file pone.0040828.s002.pdf]
